# Supplementary material for: microRNA-33 controls hunger signaling in hypothalamic AgRP neurons
Source: Nat Commun. 2024 Mar 8;15:2131. doi: 10.1038/s41467-024-46427-0 (PMC10923783; doi:10.1038/s41467-024-46427-0)
Supplement: Supplementary file 1 — Supplementary Information [file 41467_2024_46427_MOESM1_ESM.pdf]

SUPPLEMENTARY INFORMATION FILE

microRNA-33 controls hunger signaling in hypothalamic AgRP neurons.

Nathan L. Price[1,2,3,4*], Pablo Fernández-Tussy[1,2,3*], Luis Varela[2,3,5,6], Magdalena P. Cardelo[1,2,3], Marya Shanabrough[2], Binod Aryal[1,2,3], Rafael de Cabo[4], Yajaira Suárez[1,2,3,7], Tamas L. Horvath[2,3,5,6,8,#] and Carlos Fernández-Hernando[1,2,3,7,#]

[1]Vascular Biology and Therapeutics Program, Yale University School of Medicine, New Haven, Connecticut, USA.
[2]Department of Comparative Medicine, Yale University School of Medicine, New Haven, Connecticut, USA.
[3]Yale Center for Molecular and System Metabolism. Yale University School of Medicine, New Haven, Connecticut, USA.
[4]Experimental Gerontology Section, Translational Gerontology Branch, National Institute on Aging, National Institutes of Health, Baltimore, MD 21224, USA.
[5]Laboratory of Glia -Neuron Interactions in the control of Hunger. Achucarro Basque Center for Neuroscience, 48940 Leioa, Vizcaya Spain.
[6]IKERBASQUE, Basque Foundation for Science, 48009 Bilbao, Vizcaya, Spain.
[7]Department of Pathology. Yale University School of Medicine, New Haven, Connecticut, USA.
[8]Department of Neuroscience. Yale University School of Medicine, New Haven, Connecticut, USA.

*These authors contributed equally to this work.
[#]**Corresponding authors**
Carlos Fernández-Hernando, PhD. 10 Amistad Street, Room 337c, New Haven, CT 06520. Tel: 203.737.2082. Fax: 203.737.2290. Email: carlos.fernandez@yale.edu.
Tamas Horvath, DVM, PhD. 310 Cedar Street, Ste BML 316, New Haven, CT 06520. Tel: 203.785.2525. Fax: 203.785-7499. Email: tamas.horvath@yale.edu.

This PDF file contains:

Supplementary Figure legends
Supplementary Figures 1, 2 and 3
Supplementary Table 1

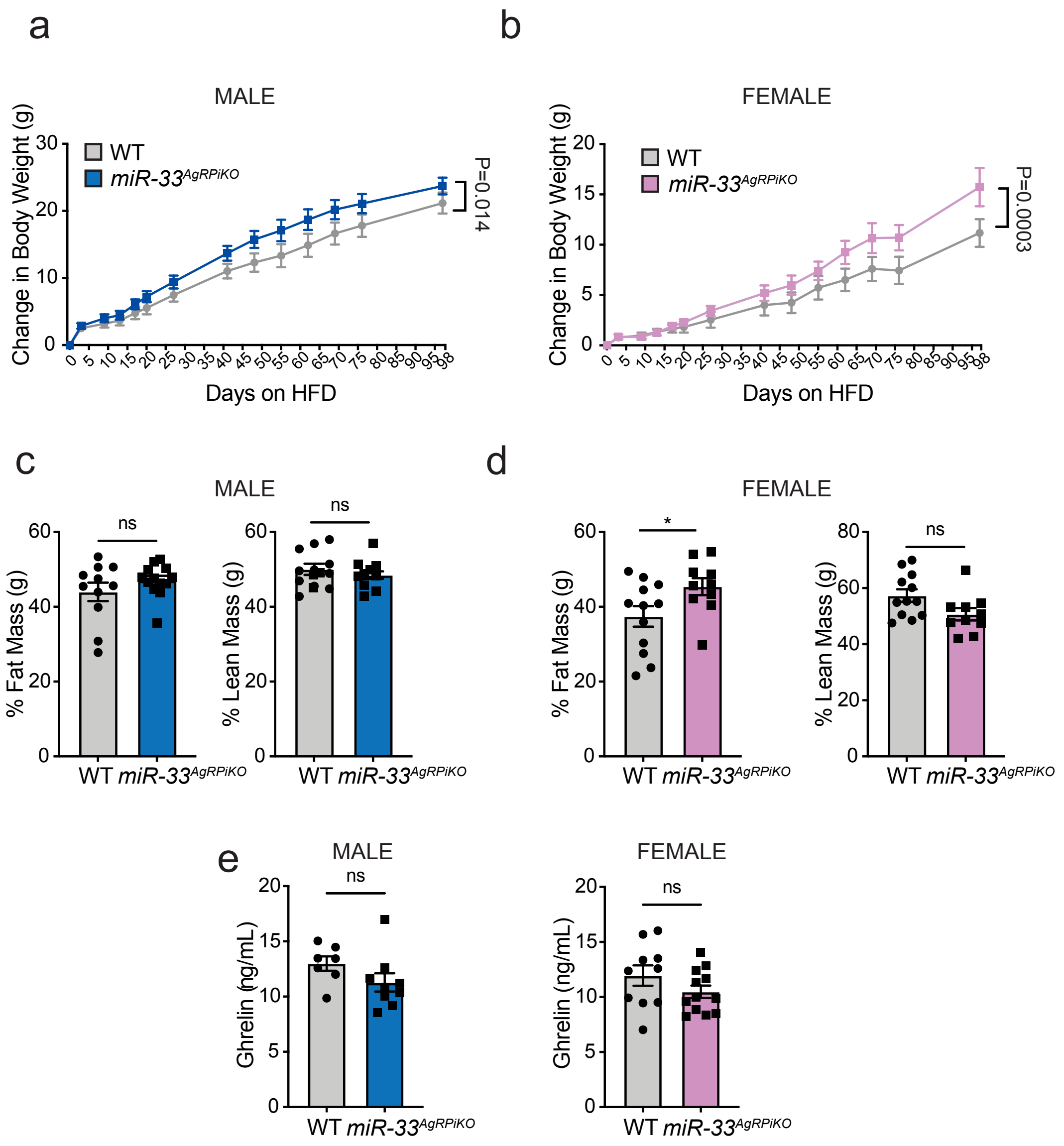

Supplementary Fig. 1: Effects of AgRP neuron specific removal of miR-33 on body weight gain and body composition. **a-b**) Change in body weight in male (**a**) and female (**b**) wildtype (WT) and *miR-33*^{AgRPcKO} mice in response to high fat diet (HFD) feeding. (n=12 WT and 14 *miR-33*^{AgRPiKO} males; n=12 WT and 10 *miR-33*^{AgRPiKO} females) **c-d**) Percent fat mass and lean mass in male (**c**) and female (**d**) WT and *miR-33*^{AgRPcKO} mice fed a HFD. (n=11 WT and 13 *miR-33*^{AgRPiKO} males; n=12 WT and 10 *miR-33*^{AgRPiKO} females). **e**) Levels of ghrelin in plasma of fasted WT and *miR-33*^{AgRPcKO} mice after HFD feeding. (n=7 WT and 9 *miR-33*^{AgRPiKO} males; n=10 WT and 12 *miR-33*^{AgRPiKO} females). All data represent mean +/- SEM. Statistical Analysis assessed by unpaired 2-sided Student *t* tests (**c-e**) or 2-way ANOVAs (**a** and **b**). Source data are provided as a Source Data file.

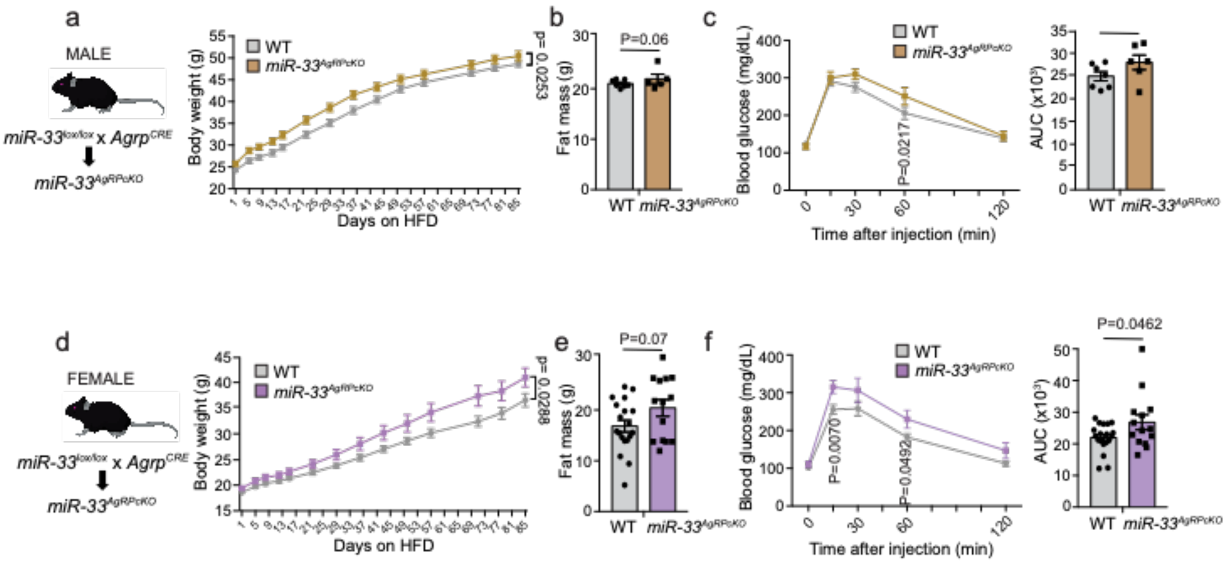

Supplementary Fig. 2: Constitutive deletion of miR-33 in AgRP neurons promotes obesity and metabolic disfunction. **a**-**f**) Body weight (**a** and **d**), fat mass (**b** and **e**) and glucose tolerance (**c** and **f**) in male (**a**-**c**) and female (**d**-**f**) wildtype (WT) and *miR-33*^{AgRPcKO} mice fed a high fat diet (HFD) (n=9-20). **a**) (n=19 WT and 9 *miR-33*^{AgRPcKO}); **b**) (n=7 WT and 5 *miR-33* ^{AgRPcKO}); **c**) (n=7 WT and 6 *miR-33* ^{AgRPcKO}); **d-f**) (n=19 WT and 14 *miR-33* ^{AgRPcKO}). Panels **a** and **d** generated using BioRender.com. All data represent mean +/- SEM. Statistical Analysis assessed by unpaired 2-sided Student *t* tests (**b,c,e,f**) or 2-way ANOVAs (**a** and **d**).

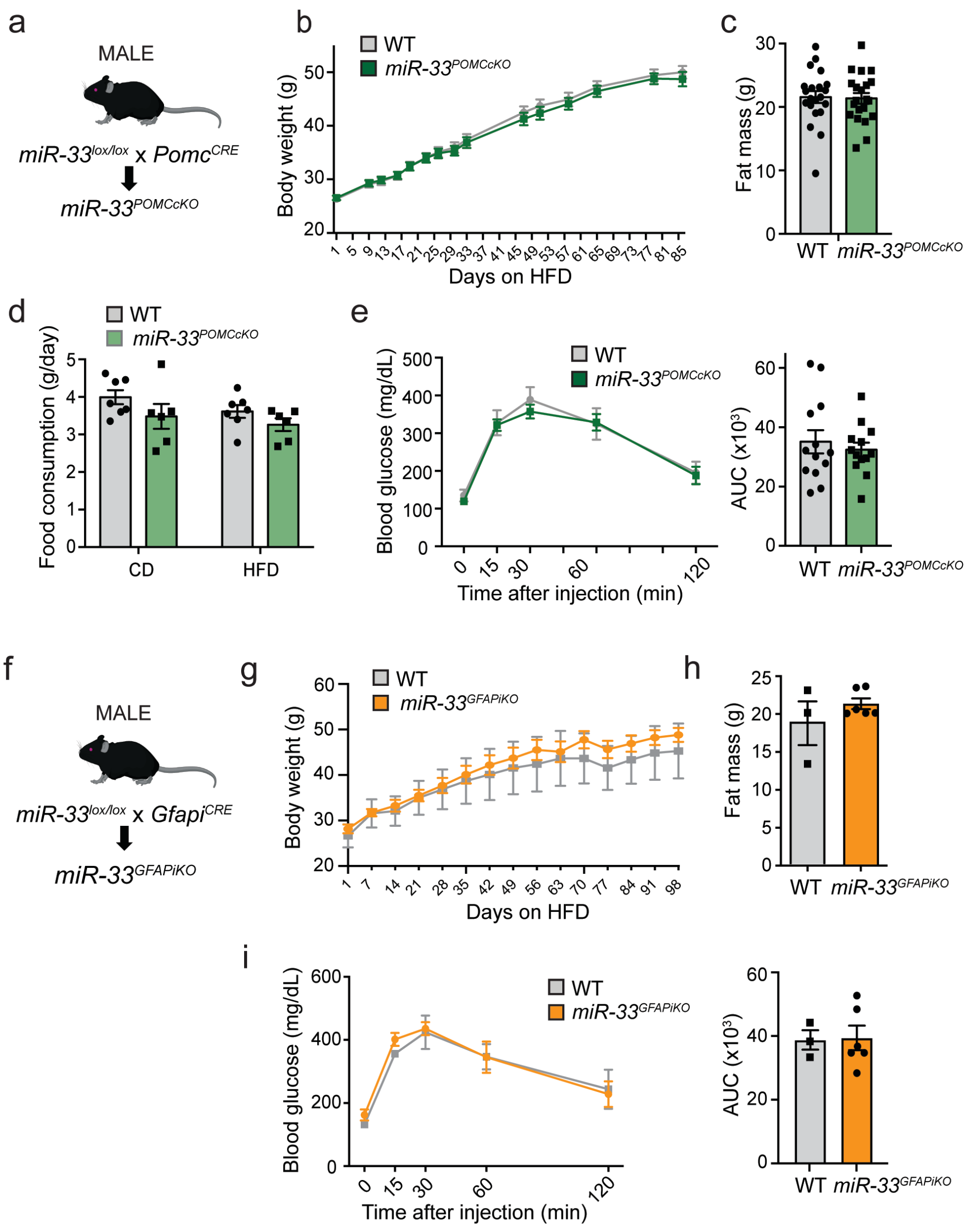

Supplementary Fig. 3: Loss of miR-33 in POMC neurons and astrocytes does not promote obesity or metabolic disfunction. a) Schematic depicting the generation of POMC neuron specific miR-33 knockout mice. Generated using BioRender.com. **b**-**c**) Body weight (**b**) and fat mass (**c**) in male WT and *miR-33*^{POMCcKO} mice fed a high fat diet (HFD) (n=21 WT and 21 *miR-33*^{POMCcKO}). **d**) Average daily food consumption in WT and *miR-33*^{POMCcKO} mice fed a chow diet (CD) or HFD (n=7 WT and 7 *miR-33*^{POMCcKO}).

e) glucose tolerance in WT and *miR-33*^{POMCcKO} mice fed HFD (n=13 WT and 12 *miR-33*^{POMCcKO}). **f**) Schematic depicting the generation of astrocyte specific miR-33 knockout mice. Generated using BioRender.com. **g-i**) Body weight (**g**), fat mass (**h**), and glucose tolerance (**i**) in male WT and *miR-33*^{GFAPiKO} mice fed a HFD (n=3 WT and 6 *miR-33*^{GFAPiKO}). All data represent mean +/- SEM. Statistic Analysis assessed by unpaired 2-sided Student *t* test (**c-e, h-i**) or 2-way ANOVAs (**b** and **g**).

Supplementary Table 1. Metabolic Cage Analysis Statistics.

		**Full Day**		**Light**		**Dark**	
Effect	Analysis	Mass	Group	Mass	Group	Mass	Group
Oxygen Consumption (ml/hr)	*GLM*	<0.001 ***	0.0445 *	<0.001 ***	0.0070 **	0.0053 **	0.148
Carbon Dioxide Production (ml/hr)	*GLM*	<0.001 ***	0.0297 *	<0.001 ***	0.0012 **	0.0152 *	0.219
Energy Expenditure (kcal/hour)	*GLM*	<0.001 ***	0.0409 *	<0.001 ***	0.0050 **	0.0064 **	0.1586
Respiratory Exchange Ratio	*ANOVA*		0.0385 *		0.0025 **		0.9508
Locomotor Activity (beam breaks)	*ANOVA*		0.2644		0.0895		0.4505
Ambulatory Activity (beam breaks)	*ANOVA*		0.3016		0.103		0.4882
