# Supplementary material for: microRNA-33 controls hunger signaling in hypothalamic AgRP neurons
Source: Nat Commun. 2024 Mar 8;15:2131. doi: 10.1038/s41467-024-46427-0 (PMC10923783; doi:10.1038/s41467-024-46427-0)
Supplement: Supplementary file 3 — Description of Additional Supplementary Information [file 41467_2024_46427_MOESM3_ESM.pdf]

Description of additional supplementary files

File Name: Supplementary Data 1.

Description: Canonical Pathway Analysis regulated in AgRP neurons from wildtype (WT) and *miR-33*^{AgRPiKO} mice. Full list of canonical pathways determined with Ingenuity Pathway Analysis, regulated in AgRP neurons from WT and *miR-33*^{AgRPiKO} mice. Table indicates the name of the pathway, logarithmic *p* value, enrichment ratio, direction of *z* score and genes whose expression is altered in the pathway. Only genes with an average log2FC > 0.25 and a P < 0.05 were included for Ingenuity Pathway Analysis studies.

File Name: Supplementary Data 2.

Description: List of genes differentially expressed in AgRP neurons from wildtype (WT) and *miR-33*^{AgRPiKO} mice. List of genes with their respective *p* value, logarithmic fold change, and mean value in WT and *miR-33*^{AgRPiKO} neurons.

File Name: Supplementary Data 3.

Description: List of overlapping miR-33 targets and genes upregulated in AgRP neurons from *miR-33*^{AgRPiKO} compared to wildtype (WT) mice. List of genes upregulated in *miR-33*^{AgRPiKO} neurons overlapping with the top 1000 predicted miR-33 target genes from TargetScan7.2. Table includes respective *p* value, logarithmic fold change, and mean value in WT and *miR-33*^{AgRPiKO} neurons.
