# Supplementary material for: microRNA-33 controls hunger signaling in hypothalamic AgRP neurons
Source: Nat Commun. 2024 Mar 8;15:2131. doi: 10.1038/s41467-024-46427-0 (PMC10923783; doi:10.1038/s41467-024-46427-0)
Supplement: Supplementary file 7 — Reporting Summary [file 41467_2024_46427_MOESM7_ESM.pdf]

nature portfolio

Corresponding author(s): Carlos Fernandez-Hernando

Last updated by author(s): Feb 5, 2023

Reporting Summary

Nature Portfolio wishes to improve the reproducibility of the work that we publish. This form provides structure for consistency and transparency in reporting. For further information on Nature Portfolio policies, see our Editorial Policies and the Editorial Policy Checklist.

Statistics

For all statistical analyses, confirm that the following items are present in the figure legend, table legend, main text, or Methods section.

n/a	Confirmed	
☐	☒	The exact sample size (*n*) for each experimental group/condition, given as a discrete number and unit of measurement
☐	☒	A statement on whether measurements were taken from distinct samples or whether the same sample was measured repeatedly
☐	☒	The statistical test(s) used AND whether they are one- or two-sided<br>*Only common tests should be described solely by name; describe more complex techniques in the Methods section.*
☐	☒	A description of all covariates tested
☐	☒	A description of any assumptions or corrections, such as tests of normality and adjustment for multiple comparisons
☐	☒	A full description of the statistical parameters including central tendency (e.g. means) or other basic estimates (e.g. regression coefficient) AND variation (e.g. standard deviation) or associated estimates of uncertainty (e.g. confidence intervals)
☐	☒	For null hypothesis testing, the test statistic (e.g. *F*, *t*, *r*) with confidence intervals, effect sizes, degrees of freedom and *P* value noted<br>*Give P values as exact values whenever suitable.*
☒	☐	For Bayesian analysis, information on the choice of priors and Markov chain Monte Carlo settings
☒	☐	For hierarchical and complex designs, identification of the appropriate level for tests and full reporting of outcomes
☒	☐	Estimates of effect sizes (e.g. Cohen's *d*, Pearson's *r*), indicating how they were calculated

Our web collection on statistics for biologists contains articles on many of the points above.

Software and code

Policy information about availability of computer code

Data collection	Sections for Electron microscopy were cut on a Leica ultra-microtome, collected on Formvar-coated single-slot grids, and analyzed with a Tecnai 12 Biotwin electron microscope (FEI) with an AMT XR-16 camera.<br>Body Mass Analysis: EchoMRI<br>Glucose measurement: Contour Ultra blood glucose meter.<br>Lipids, insulin and ghrelin measurements. Absorbance: SpectraMax iD3. Molecular DEVICES.<br>Indirect calorimetry was performed using an open-circuit, indirect calorimetry system (PhenoMaster, TSE systems).<br>qRT-PCR: iCycler Real-Time Detection System (Biorad)<br>Immunofluorescence images were aquired using a Keyence BZ-X700 fluoresce microscope.<br>Single Cell RNA-Sequencing: 10X Genomics Chromium Single Cell RNA sequencing
Data analysis	Indirect calorimetry: Statistical analysis was done using CalR: A Web-based Analysis Tool for Indirect Calorimetry Experiments.<br>Fluorescent images: ImageJ (NIH).<br>Graphs/Stats: Graphpad Prism 9,<br>sc-RNA-sequencing: Sample demultiplexing, aligning reads to the mouse genome (mm10 reference genome, University of California, Santa Cruz) with Software Tools for Academics and Researchers (STAR) and unique molecular identifier (UMI) processing. Data sets were processed using CellRanger software (version 2.1.1). Seurat R package (version 4.1.0) in R (version 4.0.5). Ingenuity Pathway Analysis (Ingenuity Systems QIAGEN, content version: 47547484, 2019).

For manuscripts utilizing custom algorithms or software that are central to the research but not yet described in published literature, software must be made available to editors and reviewers. We strongly encourage code deposition in a community repository (e.g. GitHub). See the Nature Portfolio guidelines for submitting code & software for further information.

Data

Policy information about availability of data

All manuscripts must include a data availability statement. This statement should provide the following information, where applicable:

- Accession codes, unique identifiers, or web links for publicly available datasets
- A description of any restrictions on data availability
- For clinical datasets or third party data, please ensure that the statement adheres to our policy

Data Availability Statement
All data necessary to interpret the findings about the study are included within the manuscript or from corresponding author upon request. Sc-RNA-sequencing data have been deposited in the Gene Expression Omnibus database (GSE22219). Source data are provided with this paper.

Research involving human participants, their data, or biological material

Policy information about studies with human participants or human data. See also policy information about sex, gender (identity/presentation), and sexual orientation and race, ethnicity and racism.

Reporting on sex and gender	N/A. No human data reported in the study.
Reporting on race, ethnicity, or other socially relevant groupings	N/A. No human data reported in the study.
Population characteristics	N/A. No human data reported in the study.
Recruitment	N/A. No human data reported in the study.
Ethics oversight	N/A. No human data reported in the study.

Note that full information on the approval of the study protocol must also be provided in the manuscript.

Field-specific reporting

Please select the one below that is the best fit for your research. If you are not sure, read the appropriate sections before making your selection.

☒ Life sciences ☐ Behavioural & social sciences ☐ Ecological, evolutionary & environmental sciences

For a reference copy of the document with all sections, see nature.com/documents/nr-reporting-summary-flat.pdf

Life sciences study design

All studies must disclose on these points even when the disclosure is negative.

Sample size	No statistical method was used to predetermine sample size. Samples size was determined in accordance to standard practices in this field of research and based on previous analyses and experience with this or similar experimental design (PMID: 25748653; PMID: 24074868: PMID: 34890239).
Data exclusions	No data points were excluded
Replication	Experiments were replicated as indicated in the figures. Mouse studies included sufficient number to account for biological variability. Multiple techniques and models were used to validate the same finding through different experiments (e.g. scRNAseq, Ribosome pull-down, immunofluorescence, EM), each of them including 4-10 biological replicates.
Randomization	All mice in all the experimental groups were matched by age, sex and similar body weight before starting the experiments.
Blinding	The investigators were blinded during the data acquisition in the experiments. Image analysis and quantification was performed in a blinded manner by investigators.

Reporting for specific materials, systems and methods

We require information from authors about some types of materials, experimental systems and methods used in many studies. Here, indicate whether each material, system or method listed is relevant to your study. If you are not sure if a list item applies to your research, read the appropriate section before selecting a response.

Materials & experimental systems

n/a	Involved in the study
☐	☒ Antibodies
☒	☐ Eukaryotic cell lines
☒	☐ Palaeontology and archaeology
☐	☒ Animals and other organisms
☒	☐ Clinical data
☒	☐ Dual use research of concern
☒	☐ Plants

Methods

n/a	Involved in the study
☒	☐ ChIP-seq
☒	☐ Flow cytometry
☒	☐ MRI-based neuroimaging

Antibodies

Antibodies used

rb-anti-cFOS (1:2000, #sc-52-G, Santa Cruz).
donkey-anti-rabbit IgG fluor 488, 1:500, A-21206, Life Technologies.

Validation

This antibody and staining have been previously validated and extensively used in the field for similar staining (PMID: 34890239; PMID: 33848272)

Animals and other research organisms

Policy information about studies involving animals; ARRIVE guidelines recommended for reporting animal research, and Sex and Gender in Research

Laboratory animals

Generation of conditional miR-33 knockout mice (miR-33loxP/loxP) was accomplished with the assistance of Cyagen Biosciences Inc. The success of this approach has been verified by Southern blotting and confirmed by PCR based genotyping using specific primers. To generate cell type specific miR-33 knockout mice, miR-33loxP/loxP mice were crossed with AgRP-Cre or POMC-Cre strains to selectively remove miR-33 from AgRP neurons and POMC neurons, respectively. To remove miR-33 from astrocytes, miR-33loxP/loxP mice were crossed to GFAP-ERT2Cre mice. Induction of CRE in this model was achieved by intraperitoneal injection of tamoxifen (100 mg/kg body weight) into miR-33flox/flox/GFAP-ERT2Cre and control mice for 5 consecutive days. Experimental cohorts were produced by crossing miR-33loxP/loxP males that were hetertozygous for Cre with miR-33loxP/loxP females lacking Cre to generate control (miR-33loxP/lox,Cre-) and cell type specific miR-33 knockout animals (miR-33loxP/loxP,Cre+) These Cre-strains were kindly provided by the laboratory of Tamas Horvath. As leakiness has previously been reported for the constitutive AgRP-cre line, we pre-screened mice generated with this model for non-specific miR-33 excision and limited our analysis to animals that did not show excision in other tissues.
To induce the removal of miR-33 from AgRP neurons of adult animals, we crossed our miR-33loxP/loxP mice to a recently developed AgRP-ERT2Cre-Ai14 strain that was also provided by the Horvath lab. First, mice expressing a tamoxifen-inducible Cre recombinase (CreERT2) in cells expressing AgRP (AgrpCre:ERT2)48 were crossed with Rosa26-lox-stop-lox-tdTomato (Ai14; cre-recombinase-dependent expression) mice (Ai14 reporter mice; stock #007914; The Jackson Laboratory, Bar Harbor, ME, USA) to label AgRP-expressing cells. AgRP-ERT2Cre-Ai14 mice have AgRP-expressing cells with the expression of tdTomato by tamoxifen administration. No observation of AgRP-tdTomato expression was found in the absence of tamoxifen administration, indicating that recombination was strictly dependent upon tamoxifen-induced Cre recombinase activation49.miR-33loxP/loxP were crossed with AgRP-ERT2Cre-Ai14 mice to generated mice that were heterozygous for the miR-33 inducible knockout allele, with and without AgRP-ERT2Cre-Ai14. These mice were then intercrossed to generate mice homozygous for the miR-33loxP/loxP or WT allele with AgRP-ERT2Cre-Ai14. Induction of CRE in this model was achieved by intraperitoneal injection of tamoxifen (100 mg/kg body weight) into miR-33flox/flox/AgRP-ERT2Cre-Ai14 mice that have been fasted for 18 hours to induce expression of AgRP. 6 hours later, these mice are refed and allowed to recover for at least 48 hours. This process is then repeated for a total of 5 times, resulting in efficient induction of CRE in AgRP neurons. AgRPERT2Cre-Ai14 mice that do not have the floxed miR-33 allele serve as controls for these studies and undergo the same tamoxifen induction regime. the RiboTag mouse model [B6J.129(Cg)-Rpl22tm1.1Psam/SjJ] was obtained from the Jackson Laboratory (Stock No. 029977) and crossed with miR-33flox/flox/AgRP-ERT2Cre-Ai14 or miR-33wt/wt/AgRP-ERT2Cre-Ai14 mice. All strains generated were backcrossed in to the miR-33loxP/loxP to ensure the C57BL/6J genetic background before the experiments. For diet induced obesity experiments, mice were fed a standard chow diet for 8-10 weeks after which were either switched to a high fat diet containing 60% calories from fat (D12492; Research Diets Incorporated, New Brunswick, NJ, USA) for 8-20 weeks or maintained on chow diet. Mice used in all experiments were sex and age matched and kept in individually ventilated cages in a pathogen-free facility. All of the experiments were approved by the Institutional Animal Care Use Committee of Yale University School of Medicine.

Wild animals

No wild animals were used in this study

Reporting on sex

Findings reported in the manuscript apply to both male and female mice as mice sex is indicated in each experiment. Metabolic studies were both performed in male and females independently (as indicated in figures). Gene expression characterization by Ribosome immunoprecipitation and scRNA-sequencing experiments were performed in samples pulling same number of both males and females per experimental condition.

Field-collected samples

This study did not involve field-collected samples

Ethics oversight

All procedures were approved by the Institutional Animal Care and Use Committee (IACUC - Yale University)

Note that full information on the approval of the study protocol must also be provided in the manuscript.
